# Adverse childhood experiences and global mental health: avenues to reduce the burden of child and adolescent mental disorders

**DOI:** 10.1017/S2045796022000580

**Published:** 2022-10-17

**Authors:** C. Ceccarelli, E. Prina, O. Muneghina, M. Jordans, E. Barker, K. Miller, R. Singh, C. Acarturk, K. Sorsdhal, P. Cuijpers, C. Lund, C. Barbui, M. Purgato

**Affiliations:** 1 Global Program Expert Group on Mental Health and Psychosocial Support, SOS Children's Villages, Milan, Italy; 2 Department of Psychology, King's College London, Institute of Psychiatry, Psychology & Neuroscience, London, UK; 3 Department of Neuroscience, Biomedicine and Movement Sciences, WHO Collaborating Centre for Research and Training in Mental Health and Service Evaluation, University of Verona, Verona, Italy; 4 War Child, Amsterdam, the Netherlands; 5 University of Amsterdam, Amsterdam, the Netherlands; 6 Faculty of Education, University of British Columbia, Vancouver, Canada; 7 Research Department, Transcultural Psychosocial Organization Nepal, Kathmandu, Nepal; 8 Department of Psychology, College of Social Sciences and Humanities, Koc University, Istanbul, Turkey; 9 Department of Psychiatry and Mental Health, Alan J Flisher Centre for Public Mental Health, University of Cape Town, Cape Town, South Africa; 10 Department of Clinical, Neuro, and Developmental Psychology, Amsterdam Public Health Institute, Vrije Universiteit, Amsterdam, the Netherlands; 11 WHO Collaborating Centre for Research and Dissemination of Psychological Interventions, Vrije Universiteit, Amsterdam, the Netherlands; 12 Babeș-Bolyai University, International Institute for Psychotherapy, Cluj-Napoca, Romania; 13 Health Service and Population Research Department, King's Global Health Institute, Centre for Global Mental Health, Institute of Psychiatry, Psychology and Neuroscience, King's College, London, UK; 14 Cochrane Global Mental Health, University of Verona, Verona, Italy

**Keywords:** Adolescence, child psychiatry, evidence-based psychiatry, social factors

## Abstract

Mental disorders are one of the largest contributors to the burden of disease globally, this holds also for children and adolescents, especially in low- and middle-income countries. The prevalence and severity of these disorders are influenced by social determinants, including exposure to adversity. When occurring early in life, these latter events are referred to as adverse childhood experiences (ACEs).

In this editorial, we provide an overview of the literature on the role of ACEs as social determinants of mental health through the lenses of global mental health. While the relation between ACEs and mental health has been extensively explored, most research was centred in higher income contexts. We argue that findings from the realm of global mental health should be integrated into that of ACEs, e.g. through preventative and responsive psychosocial interventions for children, adolescents and their caregivers. The field of global mental health should also undertake active efforts to better address ACEs in its initiatives, all with the goal of reducing the burden of mental disorders among children and adolescents globally.

## Introduction

According to the recently published Global Burden of Diseases report, in 2019 mental disorders represented one of the top ten causes of disease burden worldwide (Global Burden of Disease Collaborative Network, 2022). Specifically, mental disorders represented the second largest worldwide contributor for years lived with disability and the seventh for disability-adjusted life years. This trend is consistent for children and adolescents aged 0–20. It is estimated that more than 225 million children and adolescents globally live with a mental disorder. Of these, 197 million live in low- and middle-income countries (LMICs) (88%) (Global Burden of Disease Collaborative Network, 2022). This already dire situation has been further exacerbated by the COVID-19 pandemic and the implementation of related public health and social measures, especially among younger people living in LMICs (Santomauro *et al*., 2021; WHO, 2022).

A growing body of evidence indicates that the prevalence and severity of these mental disorders are influenced by several social and economic conditions. These are referred to as social determinants of mental health and include adverse social and economic circumstances such as exposure to violence, extreme poverty and forced migration (Lund *et al*., 2018). Among social determinants of mental health, those affecting children and adolescents are of particular relevance. The first two decades of life are a vulnerable period during which exposure to adverse experiences can negatively impact emotional, behavioural and cognitive development (Lund *et al*., 2018; Patel *et al*., 2018; The Lancet Public Health, 2021). These occurrences are referred to as adverse childhood experiences (ACEs) and include exposure to sexual, physical or emotional violence, childhood neglect, household dysfunction (such as parental common mental disorders), deprivations and poverty, as well as traumatic shocks (such as parental death) (The Lancet Public Health, 2021). Again, data indicate that many of these ACEs have become more relevant since the beginning of the COVID-19 pandemic, for example, through increased rates of gender-based violence as well as violence against children (Bhatia *et al*., 2021; UN Women, 2021).

In this editorial, we aim to provide a roadmap on the literature on ACEs as social determinants of health through the lenses of global mental health. We then utilise this overview of the current state of the evidence-base in these two related fields, as a basis to highlight key avenues to reduce the burden of ACEs on mental health globally.

## ACEs as social determinants of mental health

Research highlights how exposure to ACEs is associated with a higher prevalence of mental disorders (e.g. PTSD, anxiety, depressive and conduct disorders) in childhood and adolescence that may persist into adulthood (Hughes *et al*., 2017; Baldwin *et al*., 2021; Rod *et al*., 2021). A systematic review and meta-analysis on the effects of multiple ACEs on health including 37 studies and a total of 253 719 participants, for example, reported how having multiple ACEs (up to age 16–18) is a major risk factor for several health conditions. With respect to mental health, among those exposed to at least four ACEs, associations were reported as strong for mental disorders, such as anxiety: odds ratio 3.70 (95% confidence interval 2.62–5.22) or depression: 4.40 (3.54–5.46), as well as problematic alcohol use 5.84 (3.99–8.56), and strongest for problematic drug use 10.22 (7.62–13.71) (Hughes *et al*., 2017). However, all included studies in the review used retrospective, self-reported ACEs, and may therefore be influenced by recall bias. To overcome these limitations, several registry linkage cohort studies have been carried out. Rod *et al.*, for instance, performed a cohort study including 500 000 children and youth born between 1994 and 2001 in Denmark, analysing hospital admission patterns among those exposed to adversities in childhood (Rod *et al*., 2021). Their results are in line with those reported by Hughes *et al*. (2017). Exposure to deprivation, family loss and negative family dynamics was strongly associated with adverse health outcomes, including mental and behavioural problems across ages, hazard ratio 5.17 (4.23–6.31) among those aged 0–2, 5.33 (4.84–5.87) among those 3–15 and 4.30 (3.99–4.65) among those older than 16 (Rod *et al*., 2021).

In addition to negative consequences on mental health, the literature highlights how ACEs have a strong impact on wider domains, including increased overall societal costs. A systematic review and meta-analysis analysing data from 28 European countries reported associations between individual ACEs and major overall health and financial costs on a population level. In all included countries, the ACE-attributable costs exceeded 1% of the national gross domestic product, with a median proportion of 2.6% (Hughes *et al*., 2017). This can be explained by the fact that adults exposed to ACEs are more likely to engage in health-risk behaviours and develop physical and mental illnesses that reduce their years of healthy and productive life (Hughes *et al*., 2017). In turn, this impacts the wider society, with countries suffering from a loss of productivity due to reduced workforce participation, lower revenues from taxation as well as direct healthcare costs (Schofield *et al*., 2011).

This array of negative outcomes can themselves represent ACEs for the next generations, resulting in the intergenerational transmission of adversity (Hughes *et al*., 2017). For example, mental and substance use disorders and violent behaviours arising among people exposed to adverse events in childhood can act as ACEs for their children. This could be in the form of exposure to violence, and household dysfunction due to parental mental and substance use disorders (Hughes *et al*., 2017). A rapidly expanding body of evidence focusing on the drivers of intergenerational transmission highlights the interplay of psychosocial, environmental and biological factors in this process (Bowers and Yehuda, 2016). In particular, epigenetic changes have been increasingly recognised as possible mechanisms driving the transmission of trauma effects future generations. This is thought to happen both through developmentally programmed mechanisms, resulting from stress exposure *in-utero* and in early post-natal care, as well as through pre-conception mechanisms, in which previous exposure to trauma affects the germline as well as feto-placental interactions (Yehuda and Lehrner, 2018). Breaking the vicious intergenerational cycle is therefore of central importance (Lorenc *et al*., 2020; Gondek *et al*., 2021). This can be approached, among others, also through the prevention or mitigation of the negative outcomes of ACEs as well as providing support for children and adolescents who have experienced ACEs (Sara and Lappin, 2017; Linden and LeMoult, 2022).

## ACEs research and global mental health

Although most children and adolescents live in LMICs (UN, 2022) research on ACEs is strongly centred in high-income countries (HICs). This is exemplified in the paragraphs above and particularly in the systematic review carried out by Hughes *et al.* on the effects of ACEs on health. Here, only nine out of the 44 distinct population analysed in the 37 studies included, reported samples from middle-income countries and none in low-income countries (Hughes *et al*., 2017). While results from these nine populations were overall comparable with those reported for the studies carried out in HICs, they presented proportionally more methodological shortcomings. Namely, the majority of studies focusing on LMICs assessed only a small subset of outcomes and analysed samples considered not representative (i.e. did not use a random-sample or whole-population approach) (Hughes *et al*., 2017).

This HIC-centred approach is known to be a common trend, especially in child and adolescent mental health research, that should be acknowledged, and its possible implications considered (Kieling *et al*., 2011; Fisher, 2021). Contextual elements, including culture, strongly influence many elements related to mental health, including experiences of illness and perception of symptoms and suffering, patterns of coping and help-seeking, as well as strategies for healing and intervention (Kirmayer and Swartz, 2014). This occurs with ACEs as well. Cultural institutions and practices mark the social arrangements that determine the exposure to social determinants of health (Kirmayer and Swartz, 2014), including ACEs. Looking at interpersonal violence as an example, norms relating to the perpetration and response to violence vary greatly across contexts, with some subcultures providing greater support to violent practices, in name of shared values such as that of honour (Donnelly and Ward, 2015). Generalising a knowledge base created in higher-income contexts globally, without considering local practices and knowledge, has the potential of hindering our ability to advance in research and also, in turn, of preventing access from effective care (Heim and Kohrt, 2019).

An increased focus on ACEs research in LMIC is therefore essential to shed light on the nature, impact and possible strategies to prevent and respond to these adverse events (Hughes *et al*., 2017; Purgato *et al*., 2020*a*; Wood *et al*., 2020), with the potential of relieving the global burden of mental disorders (Sara and Lappin, 2017; The Lancet Public Health, 2021). To try to bridge this research gap on ACEs in LMICs, we could capitalise on some of the knowledge gained in the field of global mental health. While research on ACEs and global mental health has developed largely independently of each other (Wood *et al*., 2020), it could be beneficial to incorporate some of the lessons learned in the latter to promote global advances in the former, and *vice versa*.

## Psychosocial interventions in ACEs prevention and response

The 2020 World Health Organization (WHO) guidelines for the promotion of mental health and prevention of mental disorders among adolescents recommend the adoption of psychosocial interventions to prevent mental disorders including those exposed to adversities such as humanitarian emergencies (WHO, 2020). These interventions have been evaluated across diverse contexts in the field of global mental health and could find application in the prevention of ACEs and treatment of their impacts.

**Fig. 1.**
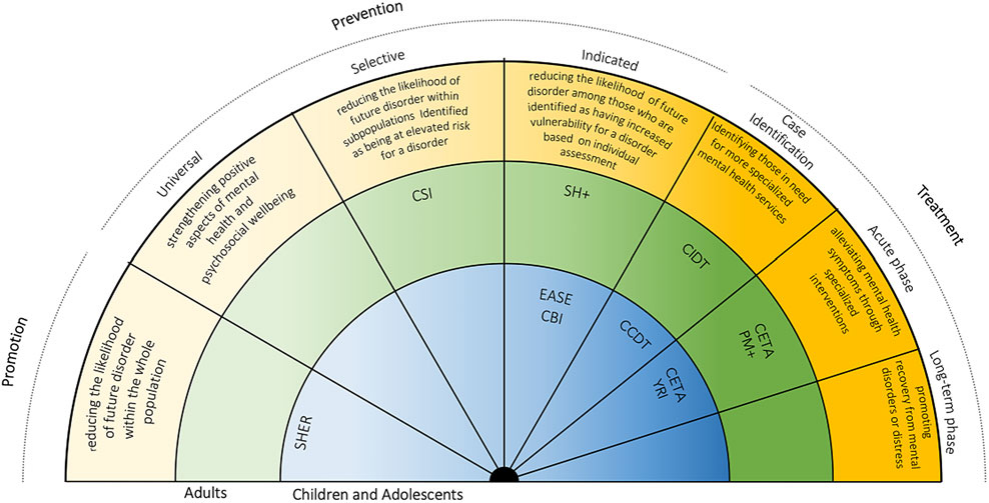
Mental health interventions continuum with a selection of psychosocial evidence-based interventions (Institute of Medicine Committee on Prevention of Mental Disorders, 1994). SEHER, strengthening the evidence base on school-based interventions for promoting adolescent health programme; CBI, classroom-based intervention; EASE, Early Adolescent Skills for Emotions; CCDT, Community Case Detection Tool; CIDT, Community Informant Detection Tool; YRI, Youth Readiness Intervention; CETA, Common Elements Treatment Approach; CSI, Caregiver Support Intervention; SH+, Self Help Plus; PM+, Problem Management Plus.

Figure 1 displays some examples of evidence-based psychosocial interventions ranging in type, target group and specific aim. These, described in Table 1, have been selected referring to findings of systematic reviews that explored the mental health promotion, prevention and treatment continuum (Tol *et al*., 2011; Purgato *et al*., 2018, 2020*b*; Barbui *et al*., 2020; Papola *et al*., 2020; Uphoff *et al*., 2020; van Ginneken *et al*., 2021) as well as guidelines by the World Health Organization and by international non-governmental organisations working with children and adolescents (McBride *et al*., 2021; Nemiro, Hof and Constant, 2021; World Health Organization and UINICEF, 2021). The interventions, developed and tested in LMICs, share the goal of either directly improving the well-being of children and adolescents who may have been exposed to ACEs (e.g. Common Elements Treatment Approach) or preventing their occurrence altogether, by for example fostering the well-being of adult parents or caregivers (e.g. Problem Management Plus), through scalable approaches aiming to overcome several of the challenges common in low-resource settings, such as paucity of human and financial resources.

**Table 1.** A selection of psychosocial evidence-based interventions (by name, type, population and aim) with the potential of improving the well-being of children and adolescents exposed to adverse childhood experiences (ACEs)

Intervention name	Intervention classification	Target population	Aim	Manual availability
Strengthening the evidence base on school-based interventions for promoting adolescent health programme (SEHER) (Shinde *et al*., 2020)	Promotion	Adolescents	To improve positive school climate as well as the mental health of adolescents through a multi-component school health promotion intervention.	Not available
Classroom-based intervention (CBI) (Tol *et al*., 2014)	Indicated prevention	Children	To decrease psychological symptoms and strengthen protective factors in children displaying heightened psychological symptoms.	Not available
Early Adolescent Skills for Emotions (EASE) (Dawson *et al*., 2019)	Treatment	Young adolescents	To reduce symptoms of internalising disorders, such as depression and anxiety. The intervention also includes sessions with caregivers, to build on existing strengths and improve the caregiver–child relationship.	Not available^a^
Community Case Detection Tool (CCDT) Community Informant Detection Tool (CIDT) (Jordans *et al*., 2015; van den Broek *et al*., 2021)	From promotion and universal prevention/selective prevention to indicated prevention or treatment	Children and adolescents, adults^b^	To identify children and adolescents (CCDT) or adults (CIDT) in need of mental health support and to encourage help-seeking.	CCDT available upon request to developers, CIDT available free for use
Youth Readiness Intervention (YRI) (Betancourt *et al*., 2014)	Treatment	Adolescents	To address co-morbid difficulties with externalising and internalising problems among youth facing adversity (e.g. war-affected youth).	Not available
Common Elements Treatment Approach (CETA) (Murray *et al*., 2018, 2020)	Treatment	Children, adolescents, adults^b^	To address mental health challenges (incl. CMDs, SUDs) among those who experience and/or witnessed trauma or violence, and conflict.	Not available
Caregiver Support Intervention (CSI) (Miller *et al*., 2020)	Selective prevention	Adults^b^	To improve caregiver well-being and strengthen parenting practices. In turn, this can decrease the impact of caregiver distress as an ACE, to improve the well-being of children.	Available upon request
Self Help Plus (SH+) (Acarturk *et al*., 2022)	Indicated prevention	Adults^b^	To improve well-being and prevent the development of mental disorders among adults experiencing some level of psychological distress. Decrease the impact of caregiver mental disorders as an ACE.	Available online and free for use
Problem Management Plus (PM+) (Rahman *et al*., 2019; Bryant *et al*., 2022)	Treatment	Adults^b^	To improve well-being among adults experiencing some levels of distress and living in communities affected by adversities. Decrease the impact of caregiver mental disorders as an ACE.	Available online and free for use

a Will be made available after effectiveness has been established.

b In light of improving the mental health of children, these interventions are especially relevant for parents and caregivers.

## Further avenues

The aforementioned considerations on psychosocial interventions in relation to ACEs only refer to some of the gaps in ACE research, and the overlapping areas of growth for global mental health. An important gap is epidemiological evidence, which is lacking for the population of children and adolescents exposed to ACEs living in LMICs but even more generally for the portion of children and adolescents living in LMICs. An analysis of the 2010 GBD report indicated that the representativeness of the included data was scarce. More specifically, the proportion of the target population (ages 5–17 years) represented by the available data referred to as ‘prevalence coverage’ was only 6.7%. This was particularly true for LMICs, with mean prevalence coverage of 4.5%, i.e. only one-sixth of the coverage found for HICs (Erskine *et al*., 2016). While authors of the updated 2019 GBD highlight the incorporation of a considerable amount of new epidemiological data compared to the previous report, to date no analyses on its prevalence coverage have been published; concerns regarding the quality of epidemiological data available for mental disorders remain therefore relevant (Global Burden of Disease Collaborative Network, 2022).

Furthermore, in the field of global mental health, there is consensus also on the need to further increase the magnitude and quality of evidence for psychosocial interventions in the treatment and prevention of mental disorders and promotion of mental health in children and adolescents. This applies to the general population, and is even more relevant for those exposed to unfavourable social determinants of mental health (Purgato *et al*., 2018; Papola *et al*., 2020). The 2020 WHO guidelines for the promotion of mental health among adolescents especially highlight the scarcity of evidence for interventions specifically designed for adolescents exposed to adverse events and circumstances such as violence and poverty (WHO, 2020). It is fundamental to ensure the compatibility of such interventions and assessments, with the local context and cultural factors, in their design and through adaptation (Chowdhary *et al*., 2014; Skivington *et al*., 2021). For this, developing a core set of outcomes for interventions, particularly for young populations in LMICs, could be beneficial for reducing inequity in global adolescent mental health (Kieling *et al*., 2011).

In addition to these considerations that aim to address questions related to the relevance and effectiveness of interventions, there is also a need to understand if these can be impactful in everyday practice, through considerations regarding the implementation sphere. To achieve this, Jordans and Kohrt have proposed a systematic assessment that considers alongside relevance, effectiveness and feasibility, the domain of quality of care (Jordans and Kohrt, 2020). In this model, quality of care can be assessed to understand the extent to which (a) a provider has the knowledge and skills required to deliver the intervention to the standard needed to achieve its expected goals, (b) the intervention was delivered well enough to achieve its expected goals, and (c) the participants received enough of the intended intervention content to achieve its expected goals (Jordans and Kohrt, 2020).

Finally, while thus far we focused only on psychosocial interventions, that work on response and preventive actions for ACEs, interventions modulating other social determinants of mental health, that span across the wider demographic, economic, neighbourhood and environmental domains, have gained extensive traction in the global mental health research field. Acting in the economic domain, for example, through cash transfers, has shown to be effective in increasing financial security, and playing a role in improving the mental health of adult as well as child and adolescent recipients (Zimmerman *et al*., 2021; McGuire *et al*., 2022). Along the same lines, in relation to the demographic domain, paid maternity leave has beneficial effects on the physical as well as mental health of mothers and their children (Van Niel *et al*., 2020; Shah *et al*., 2021). For a multi-domain approach to the prevention and response to ACEs to be effectively implemented, an integrated inter-sectoral approach involving coordinated and simultaneous efforts by public and private actors (e.g. judiciary, social welfare, education, health and other relevant sectors) is therefore imperative (Tol *et al*., 2014; World Health Organization, 2019).

## Conclusion

This editorial has attempted to explore the theme of ACEs in relation to child and adolescent mental health, with a focus on LMICs. We have highlighted how, despite the relevant burden of disease of mental disorders in childhood and adolescence and a consistent body of evidence suggesting the importance of early-life social determinants for their development, little research on ACEs has been carried out in LMICs. We, therefore, argue for the integration of findings from the realm of global mental health into that of ACEs by presenting a set of psychosocial interventions, for children, adolescents and their caregivers, that can act as preventative and responsive means to reduce ACEs and the overall burden of mental disorders.

While this may be a first step in the direction of overcoming the current shortcomings of the highly Western-centric nature of ACEs research, we acknowledge that the field of global mental health should also undertake active efforts to better address ACEs in its initiatives. These events should be considered as central determinants of mental health and therefore integrated more systematically in the design and implementation of interventions for children and adolescents living in LMICs, posing particular attention, for example, to their detection as well as their transgenerational nature.
