# Adverse childhood experiences and global mental health: avenues to reduce the burden of child and adolescent mental disorders – CORRIGENDUM

**DOI:** 10.1017/S2045796022000646

**Published:** 2022-11-11

**Authors:** C. Ceccar, E. Prina, O. Muneghina, M. Jordans, E. Barker, K. Miller, R. Singh, C. Acarturk, K. Sorsdhal, P. Cuijpers, C. Lund, C. Barbui, M. Purgato

**Affiliations:** 1 Global Program Expert Group on Mental Health and Psychosocial Support, SOS Children's Villages, Milan, Italy; 2 Department of Psychology, King's College London, Institute of Psychiatry, Psychology & Neuroscience, London, UK; 3 Department of Neuroscience, Biomedicine and Movement Sciences, WHO Collaborating Centre for Research and Training in Mental Health and Service Evaluation, University of Verona, Verona, Italy; 4 War Child, Amsterdam, the Netherlands; 5 University of Amsterdam, Amsterdam, the Netherlands; 6 Faculty of Education, University of British Columbia, Vancouver, Canada; 7 Research Department, Transcultural Psychosocial Organization Nepal, Kathmandu, Nepal; 8 Department of Psychology, College of Social Sciences and Humanities, Koc University, Istanbul, Turkey; 9 Department of Psychiatry and Mental Health, Alan J Flisher Centre for Public Mental Health, University of Cape Town, Cape Town, South Africa; 10 Department of Clinical, Neuro, and Developmental Psychology, Amsterdam Public Health Institute, Vrije Universiteit, Amsterdam, the Netherlands; 11 WHO Collaborating Centre for Research and Dissemination of Psychological Interventions, Vrije Universiteit, Amsterdam, the Netherlands; 12 Babeș-Bolyai University, International Institute for Psychotherapy, Cluj-Napoca, Romania;; 13 Health Service and Population Research Department, King's Global Health Institute, Centre for Global Mental Health, Institute of Psychiatry, Psychology and Neuroscience, King's College, London, UK; 14 Cochrane Global Mental Health, University of Verona, Verona, Italy

In Figure 1. one of the interventions (EASE) was misplaced under ‘prevention’, instead of ‘treatment’. The labels of universal prevention and promotion were inverted. Please see the below corrected figure 1 for reference:

**Fig. 1.**
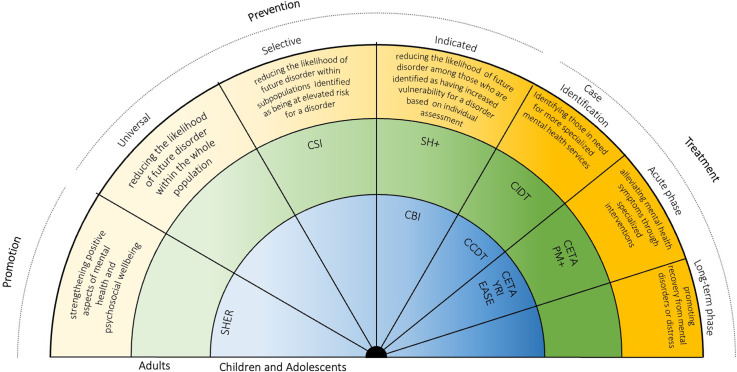
Mental health interventions continuum with a selection of psychosocial evidence-based interventions (Institute of Medicine Committee on Prevention of Mental Disorders, 1994). SEHER, strengthening the evidence base on school-based interventions for promoting adolescent health programme; CBI, classroom- based intervention; EASE, Early Adolescent Skills for Emotions; CCDT, Community Case Detection Tool; CIDT, Community Informant Detection Tool; YRI, Youth Readiness Intervention; CETA, Common Elements Treatment Approach; CSI, Caregiver Support Intervention; SH+, Self Help Plus; PM+, Problem Management Plus.
